# Modular Neural Networks for Osteoporosis Detection in Mandibular Cone-Beam Computed Tomography Scans

**DOI:** 10.3390/tomography9050141

**Published:** 2023-09-22

**Authors:** Ivars Namatevs, Arturs Nikulins, Edgars Edelmers, Laura Neimane, Anda Slaidina, Oskars Radzins, Kaspars Sudars

**Affiliations:** 1 Institute of Electronics and Computer Science, LV-1006 Riga, Latvia; arturs.nikulins@edi.lv (A.N.); edgars.edelmers@rsu.lv (E.E.); sudars@edi.lv (K.S.); 2 Department of Morphology, Institute of Anatomy and Anthropology, Rīga Stradiņš University, LV-1010 Riga, Latvia; 3 Department of Conservative Dentistry and Oral Health, Institute of Stomatology, Rīga Stradiņš University, LV-1007 Riga, Latvia; laura.neimane@rsu.lv; 4 Department of Prosthetic Dentistry, Institute of Stomatology, Rīga Stradiņš University, LV-1007 Riga, Latvia; anda.slaidina@rsu.lv; 5 Department of Orthodontics, Institute of Stomatology, Rīga Stradiņš University, LV-1007 Riga, Latvia; oskars.radzins@rsu.lv

**Keywords:** artificial intelligence, CBCT, convolutional neural network, dentistry, deep learning, osteoporosis

## Abstract

In this technical note, we examine the capabilities of deep convolutional neural networks (DCNNs) for diagnosing osteoporosis through cone-beam computed tomography (CBCT) scans of the mandible. The evaluation was conducted using 188 patients’ mandibular CBCT images utilizing DCNN models built on the ResNet-101 framework. We adopted a segmented three-phase method to assess osteoporosis. Stage 1 focused on mandibular bone slice identification, Stage 2 pinpointed the coordinates for mandibular bone cross-sectional views, and Stage 3 computed the mandibular bone’s thickness, highlighting osteoporotic variances. The procedure, built using ResNet-101 networks, showcased efficacy in osteoporosis detection using CBCT scans: Stage 1 achieved a remarkable 98.85% training accuracy, Stage 2 minimized L1 loss to a mere 1.02 pixels, and the last stage’s bone thickness computation algorithm reported a mean squared error of 0.8377. These findings underline the significant potential of AI in osteoporosis identification and its promise for enhanced medical care. The compartmentalized method endorses a sturdier DCNN training and heightened model transparency. Moreover, the outcomes illustrate the efficacy of a modular transfer learning method for osteoporosis detection, even when relying on limited mandibular CBCT datasets. The methodology given is accompanied by the source code available on GitLab.

## 1. Introduction

Artificial intelligence (AI) has the potential to revolutionize medical diagnostics by improving the accuracy, efficiency, and scalability of diagnostic decision making. One key application of AI in medical diagnostics is in the analysis of medical imaging data, such as X-rays, computed tomography (CT) scans, and magnetic resonance imaging (MRI) scans. AI algorithms, such as deep learning, can be trained on large datasets of medical images to aid in early detection or diagnosis by automatically identifying patterns and features that are indicative of specific diseases or conditions, such as lung cancer, where deep learning algorithms have been shown to be capable of identification with a high accuracy [1].

Another application of AI in medical diagnostics is in the analysis of electronic health records (EHRs) and other patient data, such as laboratory test results and vital signs. AI algorithms incorporating natural language processing (NLP) and machine learning (ML) can be trained on large datasets of EHRs to identify risk factors and commonalities which can lead to the development of specific diseases or medical conditions. For example, NLP algorithms have been used to extract information from EHRs to predict the likelihood of patients for readmission to the hospital [1]. AI can also be used to analyze genetic data and identify genetic markers that could be indicative of specific diseases or the development of certain medical conditions, which can aid in early detection and diagnosis. One such example is the use of ML algorithms to analyze genetic data and identify genetic markers associated with breast cancer [2]. While AI holds promise in revolutionizing medical diagnostics, it is not without its drawbacks. A key requirement for AI systems is the collection of extensive high-quality data for training, which can be challenging. There is also the issue of potential bias in the data used for training, which could result in skewed or erroneous predictions. Additionally, comprehending how AI models arrive at their conclusions remain complex, adding to interpretation difficulties. Although AI could notably enhance the precision and speed of medical diagnostics, leading to improved patient care and lower healthcare expenditures, it is essential to address these shortcomings through continued research. Also, responsible and ethical application of AI must be ensured.

Osteoporosis, a condition that significantly weakens the bones, is recognized by a decrease in bone mineral density and structural harm to bone microarchitecture, leading to a marked rise in the chance of fractures [3]. The World Health Organization ranks it as the second most prevalent health issue following cardiovascular disease, with one out of three women and one out of five men over 50 experiencing a fracture due to it [4]. Postmenopausal osteoporosis is the most frequently seen variant, accounting for about 62% of cases [5]. Osteoporotic fractures are often a defining disease, with a significant reduction in the quality of life, or it can even be fatal [6,7]. Consequently, it is vital to diagnose osteoporosis early and begin preventive or therapeutic measures. However, dual-energy x-ray absorptiometry (DEXA), the current primary diagnostic tool for osteoporosis, is not universally accessible and cannot be used for mass screening. Dental radiographs have shown promise in evaluating osteoporosis risk, adding value to this relatively common investigative procedure [8,9]. Nowadays, dental panoramic tomography (DPT) examinations are performed less for different surgical indications and implant planning since a sufficiently inexpensive, radiation-proportional three-dimensional examination method—cone beam computed tomography (CBCT)—is available. CBCT is now considered to be the main examination method in dental implantology, and is also widely used in other dental specialties with the number of examinations being performed increasing worldwide [10]. A CBCT examination is much more informative because it is three-dimensional, and the different jaw structures are better resolved. This suggests that changes in the quantity and quality of mandibular cortical bone detected via this examination could be even more accurate in identifying women at an increased risk of osteoporosis [11]. Our previous research demonstrated that postmenopausal women lacking teeth and with a lower bone mineral density also showed a reduction in cortical bone thickness, especially at the base of the mandible [12]. For this method to be practical in clinical settings and to avoid the necessity for additional dentist training, the development of an AI tool capable of detecting osteoporosis in CBCT scans is crucial. There are several studies regarding the use of different neural network architectures for osteoporosis screening in clinical environments which can potentially enhance the diagnosis at early stages for patients with untreated osteoporosis [8,13,14].

On the validity and reliability of mandibular measurements as a biomarker for osteoporosis screening, there are studies pointing out that jaw-related fractal dimensions (FD) presented sensitivity and specificity values higher than 70%, and that sensitivity in osteoporosis screening was a better parameter than specificity [15].

The novelty of this research lies in its pioneering application of deep convolutional neural networks (DCNNs) based on the ResNet-101 [16] architecture for the detection of osteoporotic changes using cone-beam computed tomography (CBCT) images of the mandible. This represents an innovative approach to osteoporosis diagnosis, leveraging machine learning in a way that improves both diagnostic efficacy and explainability.

This study distinguishes itself by utilizing a three-stage modular strategy. Each stage has a specific purpose and contributes to the overall effectiveness of the diagnostic system. Additionally, the modular approach with functionally separated tasks provides a more robust DCNN training process and improved system explainability. This novel approach also demonstrated that even with limited datasets of mandibular CBCT images, efficient osteoporosis detection can be achieved using a modular transfer learning strategy.

This project aims to develop a deep neural network for the detection of osteoporosis risk based on maxillofacial CBCT examinations and to evaluate its performance.

## 2. Materials and Methods

This study included 188 postmenopausal female patients, aged 54–87 years (average age 69.1 ± 8.1), who were edentulous or had partial tooth loss and who underwent CBCT examinations on the same device at the Riga Stradiņš University Institute of Stomatology (Riga, Latvia) due to implant planning. Participation within the study was voluntary. This study was approved by the Ethics Committee of Rīga Stradiņš University (No. 28/05/10.2017 and No. 2-PĒK-4/336/2022). The protocol of this study followed the principles stated in the Declaration of Helsinki. The list of the software used in this research is provided in Table 1.

### 2.1. CBCT Examinations

All CBCT examinations were performed on the same device (i-CAT Next generation, KaVo Dental GmbH, Warthausen, Germany). All examinations were performed with the following parameters: 120 kVp, 5 mA, 4 s exposure time, 0.3 mm voxel size, and 160 mm (diameter) × 130 mm (height) field of view (FOV) size.

### 2.2. Dual-Energy X-ray Absorptiometry

The general bone mineral density (BMD) of the lumbar spine vertebra (L2–L4) and both femoral necks (total hip mean) was assessed using dual-energy X-ray absorptiometry (DXA) (Lunar DEXA DPX-NT, GE Medical Systems). The worst T-score reading from both areas was used. Patients were divided into three groups based on the T-score readings: normal BMD (T-score ≥ −1.0), osteopenia (T-score −1.0 to −2.5), and osteoporosis (T-score ≤ −2.5) [17]. All measurements were performed by one experienced specialist.

### 2.3. Methodology of Medical Measurements on CBCT Images and Radiological Data Acquisition

The obtained data were processed and analyzed using OnDemand3DTM (Cybermed Inc., Seoul, Korea). In the dental module system, the axial slice of the mandible in which both mental foramens were clearly visible was selected. Then, a panoramic cutting curve was manually placed in the middle of the mandible.

**Figure 1:**
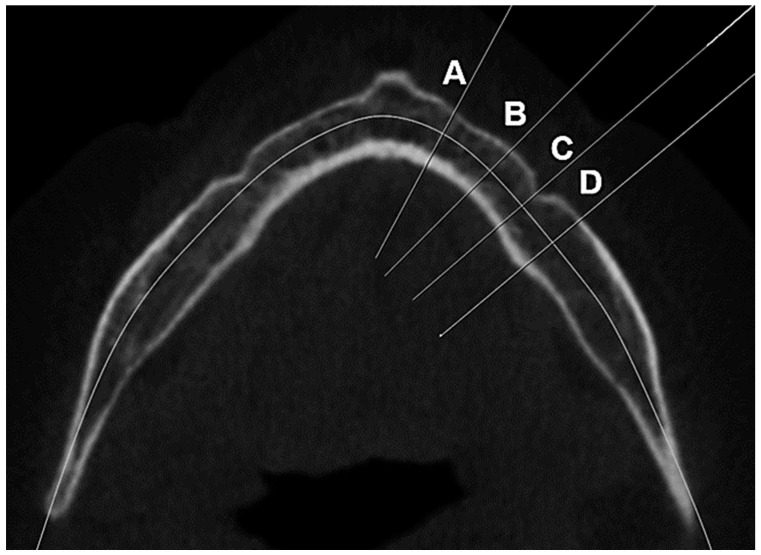
Mandibular regions where measurements were taken. (**A**) Lateral incisor region, (**B**) first premolar region, (**C**) *foramen mentale* region, and (**D**)first molar region.

In the axial slice, 4 regions were created by cutting along the mandibular arch line. A cross-sectional image of the mandible was obtained for each region: the lateral incisor region (9 mm distal from the midline), the first premolar region (6 mm anterior to the midpoint of the *foramen mentale*), the *foramen mentale* region (midpoint of the *foramen mentale)*, and the first molar region (6 mm distal to the midpoint of the *foramen mentale*) (Figure 1).

#### 2.3.1. Trabecular and Cortical Bone Volume

**Figure 2:**
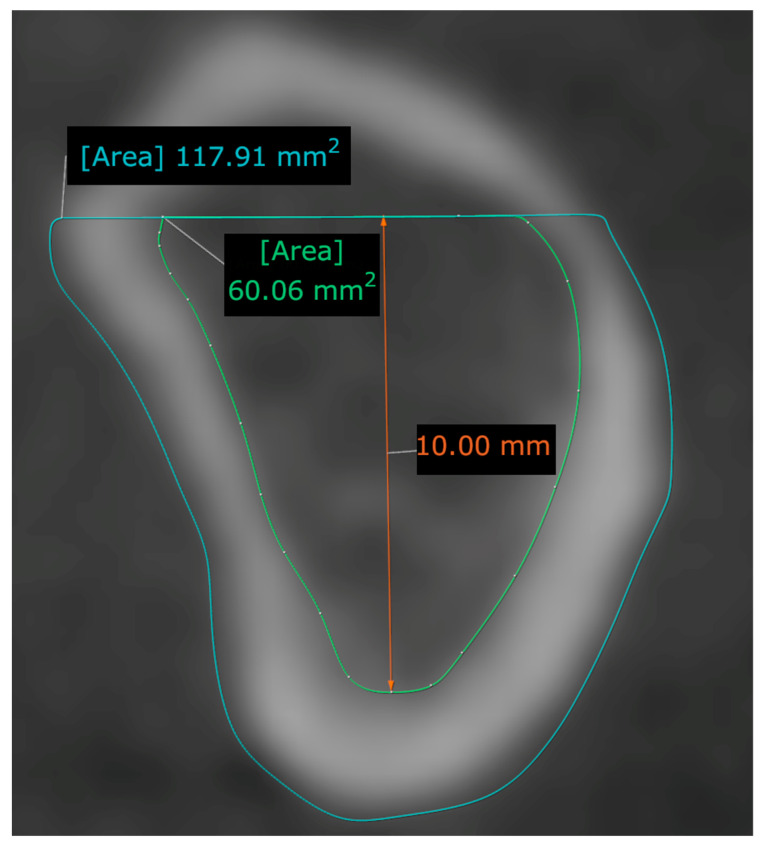
Measurement of bone area (total, trabecular, and cortical) in the basal part of the mandible.

In each region, a 10 mm long line was drawn along the longitudinal axis of the mandible in the coronal direction from the inferior border of the cortical trabecular bone border. Above the line, a perpendicular line was drawn between the vestibular and the lingual borders of the mandible. In this region, the total cross-sectional area of the jawbone, the total size of the trabecular bone area, and the difference between the two—the size of the cortical bone area—was measured according to a previously described methodology (Figure 2) [12].

#### 2.3.2. Cortical Bone Thickness

**Figure 3:**
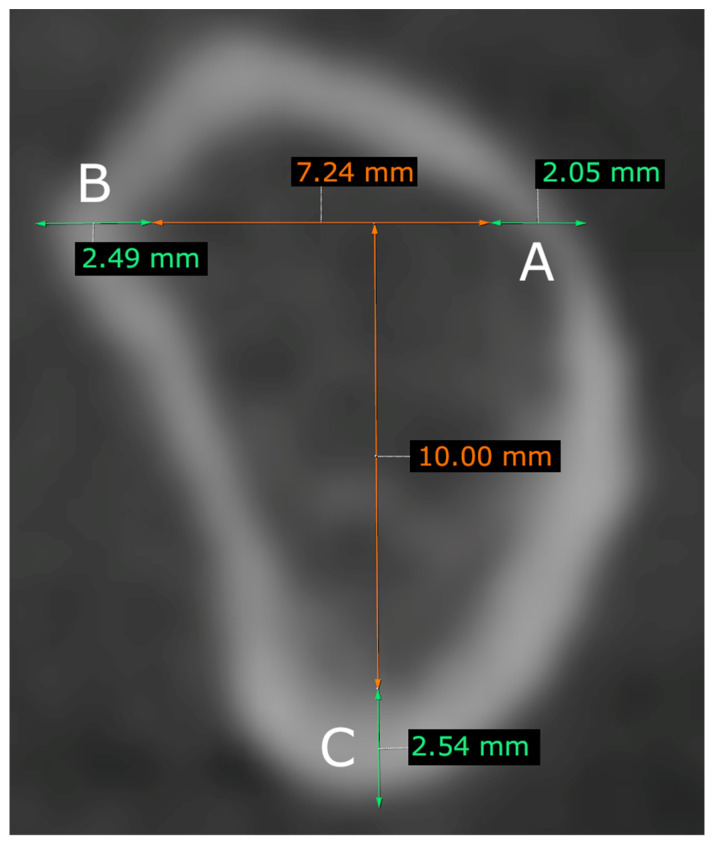
Measurements of the cortical bone thickness. (**A**) Vestibular cortical bone thickness, (**B**) lingual cortical bone thickness, and (**C**) basal cortical bone thickness.

Lingual and vestibular cortical bone thicknesses were measured on the previously drawn horizontal line, which is perpendicular to the 10 mm long line. The thickness of the basal cortical bone was measured just below the 10 mm long vertical line (Figure 3). These measurements were taken in each of the designated mandibular regions, except for the measurement of the vestibular cortical bone in the *foramen mentale* region.

#### 2.3.3. Determination of the Computed Tomography Cortical Index (CTCI)

**Figure 4:**
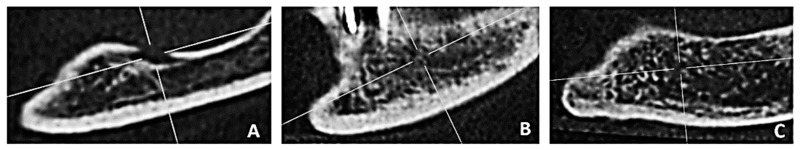
Computed tomography cortical index. (**A**) C1, the endosteal margin of the cortex was sharp and even; (**B**) C2, the endosteal margin appears semilunar defects or 1 to 2 resorption lacunae; (**C**) C3, the cortical layer formed heavy endosteal residues and was clearly porous.

In the multi-planar reconstruction (MPR) view of the Dental Module system, a modified parasagittal image of the mandible was obtained according to the method described by Koh et al. [14] The CTCI was determined by evaluating the condition of the cortical bone structure of the mandible distal from the mental foramen in the obtained parasagittal image, and it was classified into three groups:

- C1: the outer edge of the cortical bone is flat and well demarcated;
- C2: the cortical bone layer is characterized by semilunar defects or one to two resorption lacunae;
- C3: multiple (>3) resorption lacunae in the cortical layer and the inner edge of the cortical bone is markedly rough, while the cortical bone structure is markedly porous [18,19] (Figure 4).

The index was determined on both sides of the jaw, while the worst reading from both areas was considered.

#### 2.3.4. Determination of the Computed Tomography Cortical Index (CTCI) from Panoramic Reconstructed Image

In the Dental Module (Dental) system, an axial section of the mandible was selected in which both *foramina mentale* were clearly visible. A panoramic cutting curve was manually traced around the middle of the mandible. From this, a panoramic image is automatically constructed. 

**Figure 5:**
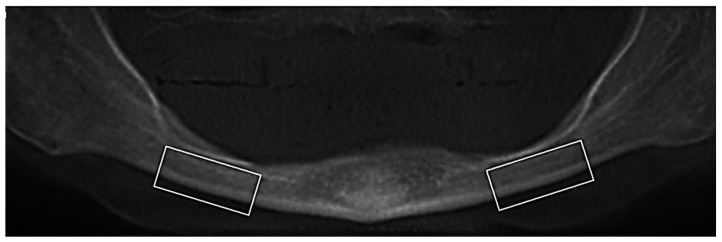
Determination of the cortical index from panoramic reconstruction image.

The panoramic image was reconstructed with two different slice thicknesses, 10.25 mm and 20.00 mm. The index was determined on both sides of the jaw (filter: HighBoost) in a region of 1.5–2 cm distal to the *foramen mentale*. The worst reading between the two was taken (Figure 5).

### 2.4. The Overall Methodology of Technical Implementation of the Deep Neural Network

The complete dataset consisted of 188 observations. The labeling process was performed following the methodology described in Section 2.1.

The list of all software is available in Table 1, while the list for hardware can be seen in Table 2.

**1. Overview of the Modular Neural Network:**

The methodology is centered on a modular neural network, an integrated system composed of two primary neural networks rooted in the ResNet-101 architecture.

1.1. **ResNet-101 Introduction:** ResNet-101 is renowned for its depth, encompassing 101 layers. Its core strength lies in its deep residual learning, effectively tackling the vanishing gradient problem. The architecture enhances performance using its distinct 3 × 3 pixel filters and 1 × 1 convolution layers. The inclusion of average pooling layers in place of fully connected ones reduces the parameter count, aiding in deeper function representation with fewer parameters [16].

**2. Radiological Data Pre-processing:**

2.1. **Data Acquisition:** Data were initially captured in the NRRD file format, a standard for storing multidimensional data like CT images.

2.2. **Data Conversion:** For compatibility and ease of processing, these NRRD files underwent a conversion process into a numpy container format, which is more amenable to data manipulation and neural network operations.

2.3. **Normalization:** Recognizing the variations inherent in radiological data, normalization was performed. This process reassigned values within each volume, pegging the minimum and maximum values to −0.5 and 0.5, respectively, ensuring uniformity and aiding the neural network in effective pattern recognition.

2.4. **Labeling:** Manual labeling, a crucial step, was executed. Experts meticulously identified and labeled the slice in the axial plane, focusing on clear visibility of both foramina mentale.

**3. First Classification ResNet-101 Network:**

3.1. **Network Initialization:** A neural network based on the ResNet-101 architecture was initialized with random weights and biases.

3.2. **Training:** With the labeled dataset, the network underwent a supervised training regimen, refining its weights and biases.

3.3. **Output Analysis:** The post-training scenario presented the network’s ability to classify slices into two categories, “yes” (presence of mandibular bone) or “no”. Each classification was accompanied by a probability value indicating the network’s confidence in its decision.

3.4. **Weight Storage:** To ensure repeatability and avoid retraining, all weights obtained during training were meticulously stored in a separate digital file.

3.5. **Validation:** Post-training, a separate validation set was used to evaluate the model’s performance. Key metrics like accuracy, recall, and precision were computed to assess model robustness.

3.6. **Image Data Processing:** A comprehensive image dataset was fed into the network. For each slice, the network computed and assigned a probability coefficient, indicating the likelihood of that slice containing the mandibular bone.

3.7. **Optimal Slice Identification:** An algorithmic approach was employed to sift through probability coefficients, selecting the slice with the highest value, earmarking it for subsequent analyses.

In the initial stage, we utilize the ResNet-101 neural network model for a classification task. We sourced the ResNet-101 model from PyTorch’s official library. Modifications were carried out in the final layers of the ResNet-101 model to better suit our desired outcomes. The outcomes were categorized into two classes: “correct slice” and “incorrect slice”.

The goal at this stage is to identify the accurate jaw slice from MRI digital images. The neural network vertically scans all slices and selects the one representing the jaw. As we proceed to future phases, this specific slice becomes crucial for making an orthogonal cut in a designated jaw area. Essentially, slices showcasing the jaw are deemed valid, serving as a benchmark for subsequent stages. Theoretically, only a single slice is the correct one, showcasing two mandibles. However, in practical scenarios, one or two slices immediately above or below may also be considered as valid. The slice with the highest probability is the one we extracted.

Regarding model adjustments, we replaced the initial convolution layer with another convolutional layer. This new layer has an input of 1 and 64 output channels, with a kernel size of (7.7), a stride of (2.2), padding of (3.3), and no bias. The final layer, known as the fully connected layer (fc), has been substituted with a series of layers: Linear(2048, 512), ReLu, Dropout(0.2), Linear(512, 2), and a softmax layer. These fc layers facilitate classification.

For optimization, we have chosen the SGD optimizer with a learning rate of 0.001, and utilized the cross-entropy loss metric. Additionally, the data need to be in the .npy format for processing (Algorithm 1).
**Algorithm 1: Modification of the initial convolution layer***  from torchvision.models import resnet101* *  model_transfer = resnet101(pretrained=True)* *  # (conv1): Conv2d(3, 64, kernel_size=(7, 7), stride=(2, 2), padding=(3, 3), bias=False)* *  model_transfer.conv1 = nn.Conv2d(1, 64, kernel_size=(7, 7), stride=(2, 2), padding=(3, 3), bias=False)* *  classifier = nn.Sequential(nn.Linear(2048, 512),* *  nn.ReLU(),* *  nn.Dropout(0.2),* *  nn.Linear(512, 2),* *  nn.Softmax()* *  )* *  model_transfer.fc = classifier* *  16:24* *  cel = torch.nn.CrossEntropyLoss()* *  optimizer = optim.SGD(model.parameters(), lr=0.001)*

**4. Second Regression ResNet-101 Network:**

4.1. **Objective Setting:** This network was specifically designed to detect seven pivotal points on the identified slice, five associated with the mandibular bone line and two marking the mandibular nerve canals.

4.2. **Training Regimen:** Leaning on the previously labeled data, the network is trained, with a focus on accurately detecting these seven points.

4.3. **Post-Training Analysis:** Once trained, the network’s capabilities are harnessed to determine the mandibular bone line and calculate the perpendicular intersection zone of the mandibular bone.

The second phase focuses on a regression task. For this, we leveraged the advanced ResNet-101 architecture. The input to this network is the correct MRI slice identified in the initial stage. The model yields 14 output classes. Each of these classes provides a value representing either the x or y coordinate of a point, with a total of 7 points. Adjustments have been carried out in the classification layer similar to the first phase, but in this case, the subsequent linear layer is designed to output 14 classes, detailing the x and y coordinates for each respective point (Algorithm 2).
**Algorithm 2: Modification of the classification layer***  from torchvision.models import resnet101* *  model_transfer = resnet101(pretrained=True)* *  # (conv1): Conv2d(3, 64, kernel_size=(7, 7), stride=(2, 2), padding=(3, 3), bias=False)* *  model_transfer.conv1 = nn.Conv2d(1, 64, kernel_size=(7, 7), stride=(2, 2), padding=(3, 3), bias=False)* *  classifier = nn.Sequential(nn.Linear(2048, 512),* *             nn.ReLU(),* *             nn.Dropout(0.2),* *             nn.Linear(512, 14))* *  model_transfer.fc = classifier* *  cel = torch.nn.CrossEntropyLoss()* *  optimizer = optim.Adam(model.parameters(), lr=0.001)*

**5. Third Stage—Thickness Analysis:**

5.1. **Thickness Computation:** Utilizing the data from the earlier stages, algorithms were developed to precisely calculate the thickness of the mandibular cortical bone, contrasting these readings against a predetermined “ground truth”.

5.2. **Bone Intersection Analysis:** Leveraging a deterministic function, perpendicular bone intersections were computed. These intersections offered insights into bone thickness, factoring in density differentials.

In the initial phase of the third stage, we must identify the mandible bone section that was cut during the second stage. Next, in this stage, we can measure the thickness of this cut bone section. This code aims to pinpoint two specific points, and the difference between these points will indicate the bone’s thickness. These points are saved in the variables “int1” and “int2”. Since bone thickness varies across different parts, we can gauge diverse thicknesses in distinct areas of the bone. Subsequently, we can compare these measurements to determine the thinnest and thickest sections of the bone (Algorithm 3).
Algorithm 3: Identifying Boundary Points*  def GetDIFF(im):* *   im = np.rot90(im, k=3)* *   ind1 = list()* *   ind2 = list()* *   # |== Find all potential points ==|* *   for i, colmn in enumerate(im):* *    if sum(colmn)!=0:* *     region = deepcopy(colmn)* *     ind1.append([np.argmax(colmn),i])  # [i, im.shape[1]-np.argmax(colmn)])* *     region[:np.argmax(colmn)]=1* *     ind2.append([np.argmin(region),i])*

## 3. Results

**Figure 6:**
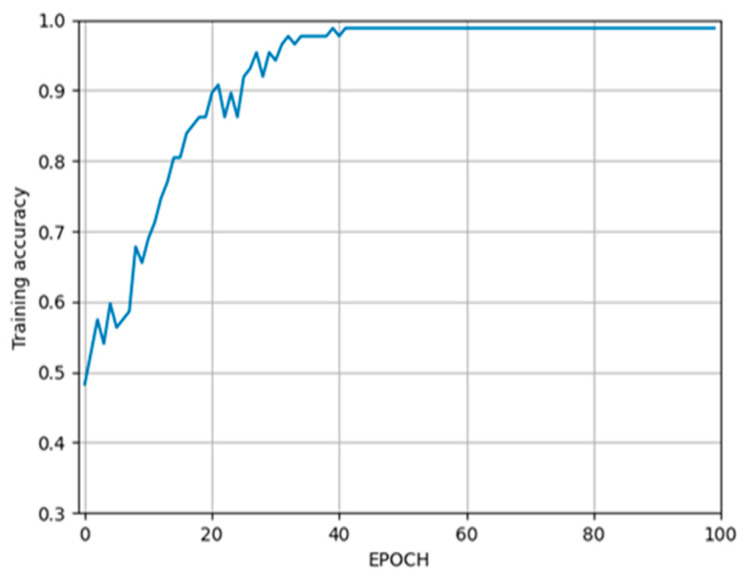
The accuracy of the training process.

**Figure 7:**
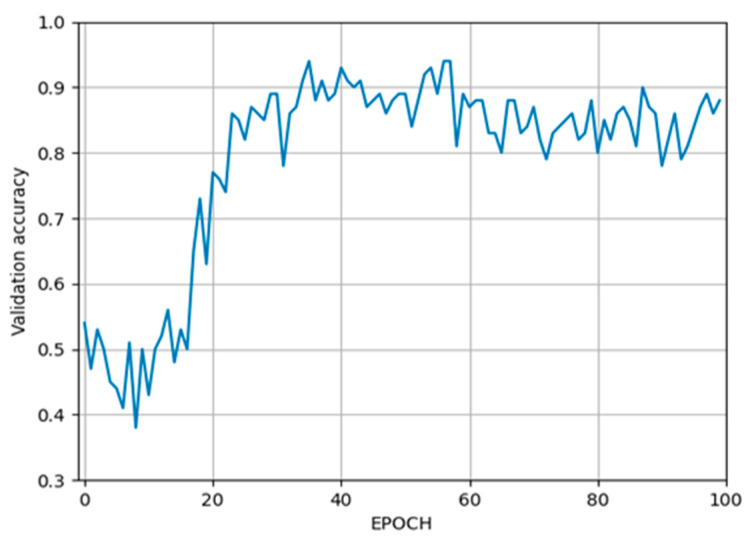
The accuracy of the validation process.

In the first stage, a classification network was created based on ResNet-101 to detect the correct slice from the CBCT scan where the whole mandibular canal is visible. The maximum accuracy (98.85%) of the training process was reached at the 39th epoch, which is plotted in Figure 6, while the accuracy of the validation process (93.99%) was reached at the 35th epoch, which is plotted in Figure 7.

The ResNet deep neural network was trained using the Stochastic Gradient Descent (SGD) optimizer with a learning rate (LR) of 0.001.

The dataset was randomly divided into training and validation sets in a ratio of 70:30.

**Figure 8:**
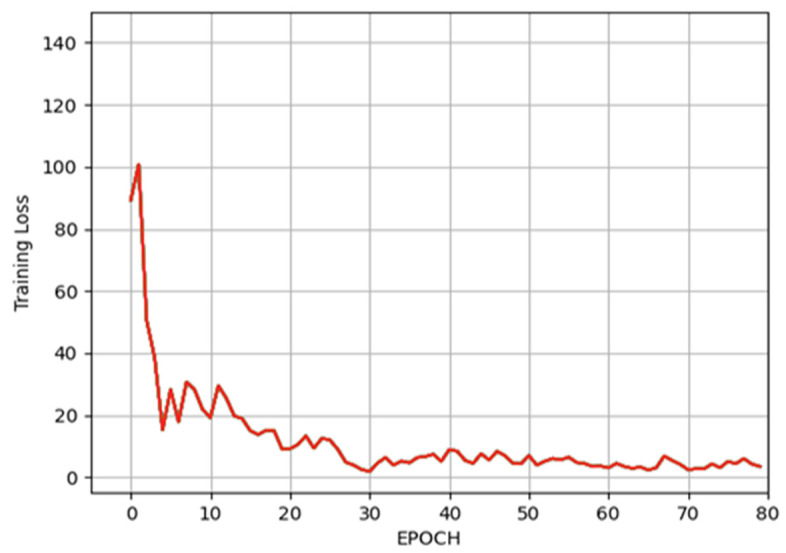
The L1 loss (mean absolute error) of the training process.

**Figure 9:**
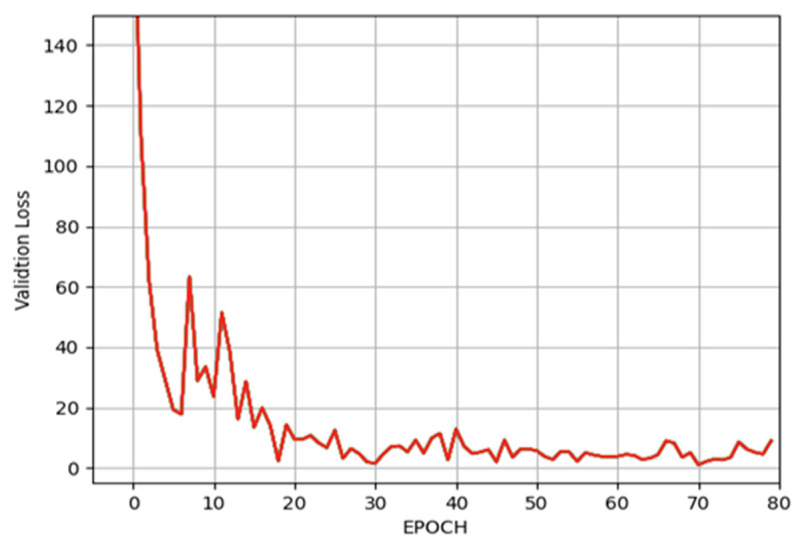
The L1 loss (mean absolute error) of the validation process.

In the second stage, a regression network based on ResNet-101 was created and trained to draw a line across the mandibular bone based on five points, and then, another line was drawn between two points which intersect the canal itself. The minimum L1 loss (showing how close the real points are to the estimated ones) of the training process reached 1.86 pixels (px) at the 30th epoch, which is plotted in Figure 8, while the minimum loss of the validation process reached 1.02 pixels at the 70th epoch, which is plotted in Figure 9.

In this stage, the ResNet deep neural network is trained using the Adam optimizer with a learning rate of 0.001.

It is possible to convert pixels to millimeters by multiplying the pixel value by a conversion factor of 0.3.

**Figure 10:**
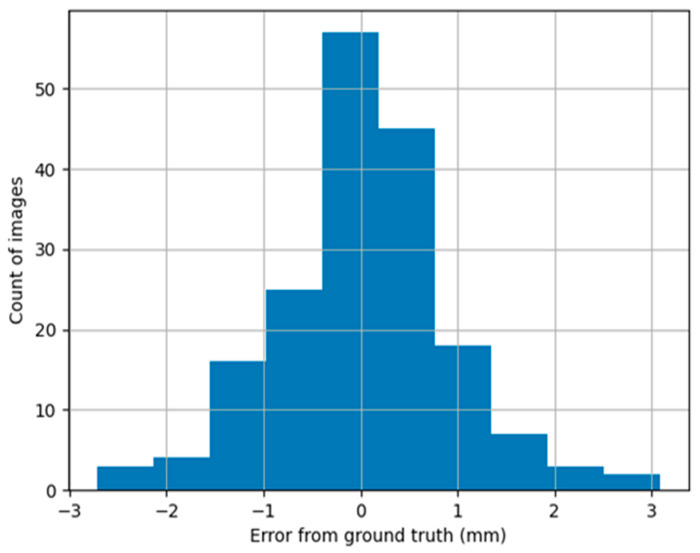
Empirical error distribution of the mandible cortical bone thickness estimation.

In the third stage, an algorithm was created to measure mandibular bone cortical thickness from a particular CBCT slice which is used to determine the presence or absence of osteoporotic changes. The empirical error distribution for this stage is depicted in Figure 10.

The mean squared error (MSE) for the estimation of mandible bone thickness was 0.8377, and the mean value was 0.018080 mm.

In Stage 3, 180 observations of mandibular bone thickness were used because not all patient data had been labeled.

The standard deviation (SD) for the true mandibular bone thickness was 0.9609 mm, and the mean value was 2.9712 mm.

## 4. Discussion

This paper outlines an algorithm that utilizes a deep convolutional neural network model to classify CBCT images of mandibular bone tissue for indications of osteoporotic changes. The neural networks suggested herein have demonstrated a commendable classification accuracy of 93.99% in Stage 1, a validation L1 loss approximating 1.02 px in Stage 2, and a Mean Squared Error (MSE) of 0.8377 when estimating mandibular cortical bone thickness in Stage 3, which holds medical significance. This serves as a strong indicator that appropriately devised and trained convolutional neural networks have the potential to lay the groundwork for the creation of an efficacious diagnostic technique for osteoporosis via the classification of mandibular bone tissue CBCT images [20]. The next steps of our research involve establishing a comprehensive diagnostic system for osteoporosis, grounded in CBCT images of the mandible and other bones impacted by this condition [21]. Turning this prototype diagnostic system into a practical application would necessitate testing it on a larger cohort of patients, along with enhancing the classification accuracy and sensitivity of the algorithm [22].

In terms of limitations, the limited size of the patient sample, consisting of only 188 participants, might reduce the generalizability of the findings to broader populations. For this model to be effectively utilized in clinical settings, it needs to be validated on larger and more diverse datasets, which may capture a broader range of bone densities, age-related changes, and other possible factors affecting bone health.

Secondly, this study heavily relies on the ResNet-101 topology, which, while effective in this context, may limit the exploration of other, potentially more suitable, architectures for this specific task. While the study showed promising results, more comparisons with other DCNN topologies could potentially improve the accuracy of the detection of osteoporotic changes; however, there are no studies with a similar methodology to ours and that utilize the same ResNet-101 DCNN architecture. In addition, the difference regarding the size of the cohort group and hyperparameters should be noted [23]. Its great potential is outlined in the Guerra study about the utilization of CBTC images for radiomorphometric indices which can be used for osteoporosis diagnoses [24]. The validity of this index-based approach was proven in the De Castro study, which showed that this approach can distinguish women with osteoporosis from those with normal BMD with good sensitivity and specificity [25].

Thirdly, while the modular approach offers increased explainability and seems to improve the training process, it is also inherently complex and may introduce additional challenges and potential points of failure. Each stage is dependent on the success of the previous one, and hence, errors in the earlier stages might propagate through the pipeline and negatively affect the final results.

Finally, this study highlights the need for further refinement of this approach. As the algorithm’s performance metrics show acceptable but not exceptional outcomes, it is clear that further tuning and improvement are needed before this methodology could be widely adopted in clinical settings. Improving the accuracy and reducing the error in mandibular bone thickness measurements should be prioritized in future work.

## 5. Conclusions

In conclusion, this study illustrated the potential of deep convolutional neural networks (DCNNs) in diagnosing osteoporosis using cone-beam computed tomography (CBCT) images of the mandible. It employed CBCT scans from 188 patients and a DCNN based on the ResNet-101 architecture to analyze these scans.

This research offers an innovative three-stage modular approach for detecting osteoporosis. Each stage carries a specific functional goal, enhancing the effectiveness and explainability of the diagnostic process. Stage 1 focuses on the application of a DCNN for slice detection in the mandibular bone. Stage 2 pinpoints the locations for creating cross-sections of the mandibular bone. In the final stage, Stage 3, an algorithm calculates the thickness of the mandibular bone to identify potential osteoporotic changes.

The impressive results from each stage—98.85% training accuracy in Stage 1, an L1 loss reduction to 1.02 pixels in Stage 2, and a minimal mean squared error of 0.8377 in Stage 3—demonstrate the considerable promise of AI in osteoporosis detection and promoting healthcare. This modular transfer learning strategy proves its efficiency even with limited datasets of mandibular CBCT images.

While these findings are promising, further research is necessary to refine this approach, expand its application, and confirm its effectiveness on a larger scale. The ultimate goal is to contribute to improved patient care through the early and accurate detection of osteoporosis. As such, the modular approach presented here has the potential to facilitate a more robust DCNN training process and enhance the explainability of the AI system, making it an important avenue for future exploration.

## Figures and Tables

**Table 1:** The list of software used.

Software/Platform	Version
3D Slicer	5.2.1
Python	3.8
PyTorch	1.4
OnDemand 3DTM	1.0.10.7462
Windows	11 Pro
Ubuntu	20.04 LTS

**Table 2:** The list of hardware used.

Type	Manufacturer	Model
Central Processing Unit (CPU)	AMD	Rome 7742
Graphical Processing Unit (GPU)	Nvidia	A100
Random Access Memory (RAM)	Kingston	64 GB
Solid State Drive (SSD)	Samsung	970EVO (200 GB)

## Data Availability

https://pubgit.edi.lv/kaspars.sudars/ostak-osteoporosis-detection (accessed on 20 September 2023).
